# Detection of freshwater cyanotoxins and measurement of masked microcystins in tilapia from Southeast Asian aquaculture farms

**DOI:** 10.1007/s00216-017-0352-4

**Published:** 2017-04-20

**Authors:** Brett Greer, Ronald Maul, Katrina Campbell, Christopher T Elliott

**Affiliations:** 1 Institute for Global Food Security, School of Biological Sciences, Queens University Belfast, Stranmillis Road, Belfast, BT9 5AG UK; 2 BAM Federal Institute for Materials Research and Testing, Richard-Willstätter-Straße 11, 12489 Berlin, Germany; 3 School of Food Science, Institute of Food Chemistry, University of Hamburg, Grindelallee 117, 20146 Hamburg, Germany

**Keywords:** Harmful algal bloom, UPLC-MS/MS, Aquaculture, Microcystin, Bioaccumulation, Human health

## Abstract

Recently, there has been a rise in freshwater harmful algal blooms (HABs) globally, as well as increasing aquaculture practices. HABs can produce cyanotoxins, many of which are hepatotoxins. An ultra-performance liquid chromatography tandem mass spectrometry method was developed and validated for nine cyanotoxins across three classes including six microcystins, nodularin, cylindrospermopsin and anatoxin-a. The method was used to analyse free cyanotoxin(s) in muscle (*n* = 34), liver (*n* = 17) and egg (*n* = 9) tissue samples of 34 fish sourced from aquaculture farms in Southeast Asia. Conjugated microcystin was analysed by Lemieux oxidation to ascertain the total amount of microcystin present in muscle. Some tilapia accumulated free microcystin-LR in the muscle tissue at a mean of 15.45 μg/kg dry weight (dw), with total microcystin levels detected at a mean level of 110.1 μg/kg dw, indicating that the amount of conjugated or *masked* microcystin present in the fish muscle accounted for 85% of the total. Higher levels of cyanotoxin were detected in the livers, with approximately 60% of those tested being positive for microcystin-LR and microcystin-LF, along with cylindrospermopsin. Two fish from one of the aquaculture farms contained cylindrospermopsin in the eggs; the first time this has been reported. The estimated daily intake for free and total microcystins in fish muscle tissue was 2 and 14 times higher, respectively, than the tolerable daily intake value. This survey presents the requirement for further monitoring of cyanotoxins, including masked microcystins, in aquaculture farming in these regions and beyond, along with the implementation of guidelines to safeguard human health.

**Graphical abstract:**
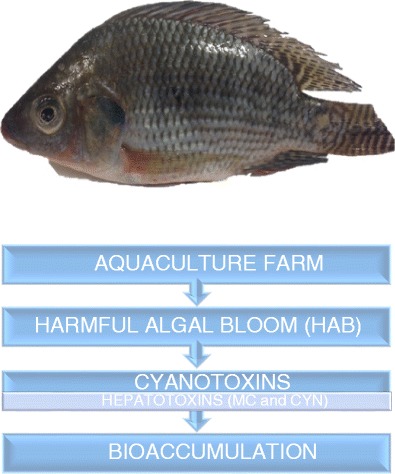
ᅟ

ᅟ

## Introduction

**Fig. 1:**
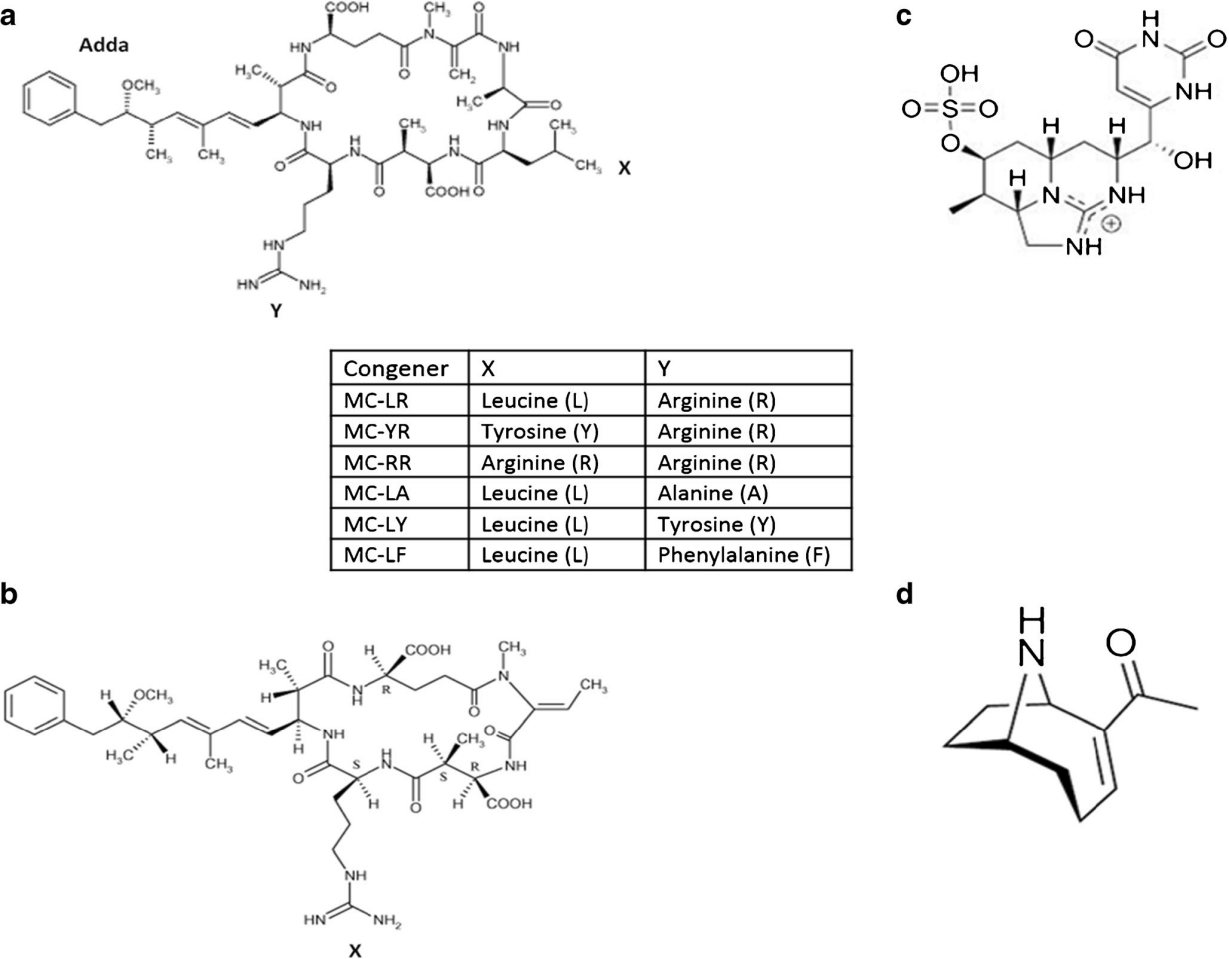
Chemical structures of the **a** cyclic heptapeptide microcystin-LR with *X* and *Y* being the variable amino acids as indicated in the accompanying table; **b** cyclic pentapeptide nodularin, with only one variable amino acid (X = arginine), the most common congener; **c** tricyclic alkaloid cylindrospermopsin (CYN); and **d** the alkaloid anatoxin-A (ATX-A)

Harmful algal blooms (HABs), found in aquatic environments across the globe, are formed when certain cyanobacterial bloom exponentially producing secondary metabolites called cyanotoxins. Approximately 40 cyanobacterial species have been identified as being capable of producing these cyanotoxins [1, 2]. One such cyanobacterium, *Microcystis aeruginosa*, is known to produce the cyanotoxin microcystin (MC) of which around 100 different congeners have been characterised to date and which has widespread occurrence globally [3]. Another producer, *Cylindrospermopsis raciborskii*, is rapidly spreading with its bioactive metabolite cylindrospermopsin (CYN) reported in several countries [4]. Of particular importance are the hepatotoxins comprising the cyclic peptides (MCs and nodularin (NOD)), the tricyclic alkaloid (CYN) as well as the potent neurotoxin (anatoxin-a (ATX-A)), with the structures outlined in Fig. 1 [2, 5]. The MC congeners are cyclic heptapeptides, with structural variations coming from differences in the amino acids at positions 2 and 4 within the cyclic body of the molecule.

The MCs and NOD are potent hepatotoxins, known to cause liver failure by their inhibition of protein phosphatase type 1 (PP1) and protein phosphatase type 2A (PP2A) in liver cells [6]. This happens through the formation of a non-covalent bond between the β-amino acid 3-amino-9-methoxy-2,6,8-trimetyl-10-phenyldeca-4,6-dienoic acid (Adda) side chain and the glutamyl carboxyl, followed by a covalent, irreversible bond formed between the *N*-methyldehydroalanine (Mdha) group of the toxin and cysteine residue(s) of the protein phosphatase. There is also evidence indicating MC-LR as a tumour promoter, as in 2010, it was classified as a group 2B carcinogen by the IARC, whose evaluation deemed it as possibly being carcinogenic to humans [7].

Another of the cyanotoxins, CYN, is a known inhibitor of protein synthesis across multiple organ systems such as the liver and kidneys, with the liver being the primary target organ for bioaccumulation and toxicity. Because of its impact on other organs, CYN can also be regarded as a general cytotoxin, with its mode of action mediated through inhibition of protein synthesis and cytochrome P450, as well as genotoxicity caused by DNA fragmentation. Furthermore, it has also been suggested that CYN could be classified a potential carcinogen [8].

The neurotoxin, ATX-A, interferes with transmission of nerve impulses as it mimics the neurotransmitter acetylcholine. It acts as a potent cholinergic blocking agent and can compete with acetylcholine for the nicotinic and muscarinic receptors. Exposure results in overstimulation of the receptors leading to persistent muscle stimulation and subsequent death due to muscular paralysis and respiratory failure [2]. The ingestion of cyanotoxins through drinking water has been implicated in the poisonings of both humans and animals [2, 9], with human poisonings documented across continents such as North and South America, Europe and Asia [3]. One incident, in particular, resulted in numerous human fatalities when patients undergoing renal dialysis were exposed to MCs in Caruara, Brazil [10].

Increasing reports of the bioaccumulation of cyanotoxins in freshwater *seafood* have been extensively reviewed [11, 12] with the warning that human exposure through this route may be underestimated. A study on fishermen subsisting from Lake Chaohu in China showed MCs in the water, accumulation of these toxins in the fish and subsequent uptake into the serum of the fishermen through fish consumption. Additional investigation of liver function enzymes indicated hepatocellular damage in these men [13]. There is also evidence to suggest that the high levels of liver cancer in regions of Southeast Asia and China could be linked to the drinking of surface water, where cyanobacteria and possibly cyanotoxins are abundant [7, 14]. More recently, it has been shown that MC-LR may promote the migration and invasion of colorectal cancer and has also been implicated in renal function impairment through consumption of MC-LR-contaminated water and food [15, 16].

Linked to the spread of these freshwater toxins globally in aquatic environments is the increase in inland aquaculture, contributing almost 65% to the world’s farmed food fish production between 2003 and 2013. The global aquaculture production of the Nile tilapia (*Oreochromis niloticus*) contributed almost 3.5 million tonnes in 2013 and is becoming one of the most farmed fish in the world. Since 2008, Asia has produced more fish through aquaculture with its share in total production reaching almost 90% in 2014. Almost all fish produced by aquaculture is used for direct human consumption with the remaining utilised for the manufacture of fishmeal and fish oils [17].

Due to the intensive use of freshwater resources, lake and pond water is becoming increasingly polluted with the resulting increase in nutrient load giving rise to HABs [18]. Examples of this are occurrences of dense HABs in many Chinese lakes, such as Lake Taihu, Lake Chaohu and Lake Dianchi, which have increased in frequency in recent years [19]. MCs have also been detected in 54% of aquaculture ponds in Zaria, Northern Nigeria, with several having MC levels above the World Health Organization (WHO) limit of 1 μg/L [20] as well as being identified in a freshwater fishing lake in Mexico, characterised by a persistent HAB containing MCs [21]. Aquaculture species can be exposed to cyanotoxins through the aquatic food chain and can accumulate in their tissues facilitating transfer through the food chain, although their bioaccumulation or biomagnification is ambiguous depending on the species. MCs, and to a lesser extent CYN, have been shown to bioaccumulate in invertebrates such as bivalves and also in fish [11]. Accumulation of MCs has been shown in the muscle (edible) tissue of various species of fish within natural systems [19, 22, 23]. Note that, in some cases, fish can be eaten whole and, therefore, the viscera can contribute to the overall toxicity. There have also been reports of CYN in natural freshwater systems such as in the muscle of crayfish [24] and tegogolos snails from a freshwater lake in Mexico [21]; however, although CYN was detected in the viscera of the rainbow fish *Melanotaenia eachamensis* in a freshwater system [24], there were no reports of this toxin in the edible parts of any fish species until a study by Berry et al. [25] showed the apparent accumulation of CYN in fish muscle across several species of fish in a freshwater lake, albeit at low levels of <1 μg/kg wet weight (ww) [25].

MCs can accumulate in fish tissues as *free* toxin or can be found as bound/conjugated (masked) through their binding to PP1 and PP2A in certain tissues as outlined above. Once bound, they cannot be detected by conventional extraction techniques. Instead, the use of Lemieux oxidation, a technique known to produce 2-methyl-3-methoxy-4-phenylbutyric acid (MMPB) by oxidation of the Adda side chain found in each MC congener [26], allows for the total level of MC to be calculated. This value consists of both free and covalently bound MC, allowing the total exposure to MCs to be ascertained.

Although hepatotoxic effects have been partially documented in humans after exposure to MCs, the effects of long-term chronic exposure to low MC levels, as well as other hepatotoxins, have not been fully investigated. The objective of this study were to (a) develop and validate a UPLC-MS/MS method for the analysis of free cyanobacterial toxins in fish (*O. niloticus*) across three cyanobacterial classes, (b) analyse the level of total MC (and/or NOD) present by the use of Lemieux oxidation and (c) assess the potential risk to human health through consumption of fish (*O. niloticus*) grown in commonly employed pond-based aquaculture farms in SE Asia.

## Materials and methods

### Materials

MCs (MC-LR, MC-YR, MC-RR, MC-LA, MC-LY and MC-LF) and NOD standards were purchased from Enzo Life Sciences, Ltd. (UK), CYN was obtained from n’Tox (France) and ATX-A was purchased from the National Research Council, Canada. MMPB was purchased from Wako Pure Chemical Industries, Ltd. (Japan). CYN N^15^-labelled internal standard (N^14^ → N^15^) was a gift from the Federal Institute for Materials Research and Testing (BAM), Berlin, Germany. Acetonitrile and methanol, LC-MS Chromasolv grade, dichloromethane, formic acid, trifluoroacetic acid, potassium permanganate, sodium (meta) periodate and sodium bisulfite were all purchased from Sigma-Aldrich, UK, as were the ENVI-Carb solid-phase extraction (SPE) cartridges (250 mg, 3 cm^3^). Oasis HLB and PRiME SPE cartridges (60 mg, 3 cm^3^) were purchased from Waters, Ireland. The water used was supplied from an in-house Milli-Q water system (Millipore, Ltd., UK), with conductivity and total organic content (TOC) of the water being 18 MΩ and 3 ppb, respectively.

### Standard preparation

The ATX-A standard was provided at a concentration of 4.96 μg/mL. The MCs, NOD and CYN were purchased in 100 μg quantities, with their identity confirmed by MS and purity being ≥95% as verified by HPLC, according to the certificate of analysis. Stock standards of 1 mg/mL of each cyanotoxin were carefully prepared by reconstitution of the product in the vial by the addition of 100 μL of pure methanol or, in the case of CYN, pure water. Working standards at a concentration of 10 μg/mL were then prepared by diluting stock standards (1:100 *v*/*v*) with 80% aqueous methanol (*v*/*v*) and water for CYN. The multi-toxin standard used to make the calibrants was prepared at a concentration of 0.5 μg/mL by dilution of the 10 μg/mL working standards 1:20 (*v*/*v*) and in the case of ATX-A by 1:9.92 (*v*/*v*) with 80% aqueous methanol (*v*/*v*).

### Ultra-performance liquid chromatography tandem mass spectrometry

#### Free cyanotoxin analysis

Separation and analysis of the nine cyanotoxins were carried out using an ACQUITY UPLC i-Class system coupled to a Xevo TQ-MS mass spectrometer (Waters, Manchester, UK) using multiple reaction monitoring (MRM) as detailed in Greer et al. [4], with the transitions used for the analysis outlined in Table 1. Separation was achieved on an ACQUITY UPLC HSS T3 column, 100 mm × 2.1 mm i.d., 1.8 μm particle size and 130 Å pore size (Waters, UK), in order to retain the polar CYN [27]. The two mobile phases consisted of water containing 0.1% formic acid (*v*/*v*) and the organic solvent, acetonitrile. The flow rate was set at 0.45 mL/min with the acetonitrile held at 2% for 1 min, followed by an increase to 70% over 9 min, maintained at 90% for 1 min before returning to 2% for a 1-min re-equilibration before the next injection, with the injection volume set at 1 μL.

**Table 1:** Table showing the optimised MRM transitions for the nine cyanotoxins, the N^15^-labelled cylindrospermopsin internal standard (N^14^ → N^15^) and MMPB

Compound	MRM function	Precursor ion (*m*/*z*)	Cone voltage (V)	Base fragment ion (*m*/*z*) [*Q*]	Collision energy (eV)	Qualifier fragment ion (*m*/*z*) [*q*]	Collision energy (eV)
ATX-A^a^	1	166.1	25	149	15	131.1	16
CYN	2	416.2	35	194.1	42	336.2	22
CYN (N^14^ → N^15^)	3	421.1	40	197.1	40	–	–
MC-RR	4	520.0	35	135.0	28	127.1	42
NOD	5	825.5	65	135.0	64	70.0	78
MC-LA	6	910.5	30	135.1	64	163.1	44
MC-LF	7	986.5	35	135.1	70	213.1	56
MC-LR	8	995.6	65	135.0	76	107.1	80
MC-LY	9	1002.5	35	135.2	68	163.1	62
MC-YR	10	1045.5	60	135.2	72	107.1	75
MMPB^b^	1	209.15	10	191.2	5	131.1	15
MC-LR^b^	2	496.5	25	487.4	12	128.1	25

The table is adapted and reproduced from Greer et al. [4]

*Q* = quantifier ion, *q* = qualifying ion

^a^Has a second qualifier (*q*1) used as a diagnostic ion to prevent misidentification: *q*1 = 166.1 > 42.95 (not shown in the table)

^b^Signifies the transitions used in the analysis of MMPB to determine the total amount of microcystin present

#### Total microcystin analysis

Analysis of MMPB and MC-LR was carried out on the same LC-MS/MS system as outlined in the “Free cyanotoxin analysis” section. Detection and quantification were achieved using MRM with the system operated in both ES^+^ and ES^−^, and the transitions were optimised manually (Table 1). Separation of MMPB and MC-LR was achieved on an ACQUITY UPLC BEH C18, with column dimensions of 2.1 mm × 50 mm, 1.8 μm particle size and 130 Å pore size (Waters, UK), maintained at 40 °C. The mobile phases comprised of water (0.1% acetic acid *v*/*v*) and acetonitrile with the flow rate set at 0.40 mL/min. The acetonitrile was held at 10% for 0.5 min, increased to 80% over 4 min and washed for 1 min at 90% before returning to 10% for a 1 min re-equilibration before the next injection. The injection volume was set at 1 μL.

### Extraction of cyanotoxins in water and validation of the extraction method in fish

Extraction, analysis and quantification of cyanotoxins in the 50 mL water samples collected from the five aquaculture farms were performed exactly as outlined in Greer et al. [4].

Toxin extraction and subsequent enrichment from the fish were performed according to modified methods [28, 29] and developed using 100-mg aliquots of lyophilised homogenised blank fish muscle. Extraction efficiency, reported as absolute recovery, was determined by spiking fish muscle before and after with 50 μL of a multi-toxin standard at 0.5 μg/mL, equating to a toxin level of 0.25 μg/g dry weight (dw) fish tissue, and 15 μL of the N^15^-labelled CYN internal standard (N^14^ → N^15^) at 1 μg/mL.

Validation of the method for fish muscle was performed according to the European decision 2002/657/EC concerning the performance of analytical methods for banned and controlled substances in animals used for food production [30] and was achieved using 100-mg aliquots of lyophilised blank fish muscle. The blank fish muscle used for validation consisted of pooled lyophilised muscle tissue taken from fish from aquaculture farms 1–3 and 1–4. The fish muscle and water from these farms had been shown to be negative for any of the cyanotoxins tested and would maximise variability in performance as well as allowing use of an exact matrix match.

Validation involved the extraction of 20 blank samples over three separate days followed by extraction of 20 spiked samples at 0.05 μg/g dw (addition of 10 μL of the multi-toxin standard at 0.5 μg/mL) extracted over three separate days. A seven-point extracted matrix-matched calibration curve was prepared in the range 0.01–0.5 μg/g dw. Each sample, including blanks and calibrants, was also spiked with 15 μL of the CYN Internal Standard (N^14^ → N^15^) at 1 μg/mL. Samples were extracted and analysed as outlined below, giving the inter- and intra-day precision, trueness, absolute recovery, linearity as well as CC_α_ (LOD) and CC_β_ (LOQ) values.

Briefly, samples (100 mg) were extracted by the addition of 1 mL of 75% aqueous methanol (*v*/*v*) (0.5% acetic acid *v*/*v*), vortex mixed briefly and sonicated at room temperature for 45 min before centrifugation at 16,000*g* for 15 min. The supernatant was removed and diluted fivefold by the addition of water for loading onto SPE cartridges, with samples loaded onto the Oasis HLB cartridge, and the flow through collected then passed through ENVI cartridges. Oasis HLB cartridges were washed with water and 20% aqueous methanol (*v*/*v*). Bound toxins were eluted into clean glass tubes with 2 × 1.5 mL of 80% aqueous methanol (*v*/*v*) containing 0.1% trifluoroacetic acid (*v*/*v*) for the Oasis and 2 × 1.5 mL methanol/dichloromethane (4:1 *v*/*v*) containing 5% formic acid (*v*/*v*) for the ENVI. The resulting eluents were combined, dried under a stream of nitrogen before reconstitution in 200 μL of 80% aqueous methanol (*v*/*v*) and transferred to a microvial for analysis.

The same method was applied for the extraction of toxins from liver tissue and eggs but was validated for fish muscle only due to the lack of material.

### Lemieux oxidation and extraction of MMPB

Oxidation of bound MC and subsequent extraction were performed according to a modified method [26] and developed using 50-mg aliquots of lyophilised homogenised fish muscle. A 50-mg aliquot of fish was weighed into a 50-mL round bottomed flask, and 5 mL of oxidation solution was added, comprising both potassium permanganate (KMnO_4_) and sodium (meta) periodate (NaIO_4_), each at 0.1 M, stirred at room temperature for 2 h. When the colour of the solution turned from purple (colour of KMnO_4_) to red/brown (reduced product colour), a further 3 mL oxidation solution (0.1 M) was added, turning the solution back to purple. The reaction was immediately quenched by the addition of 1 mL sodium bisulphite solution (40% *w*/*v*), and 10% sulphuric acid was added drop-wise until the pH was acidic (∼2). Samples were then centrifuged at 4500 rpm for 15 min, and the resulting supernatant was removed and loaded onto SPE columns.

Sample enrichment was achieved using Oasis PRiME cartridges, with these not requiring any conditioning. Samples were loaded onto the cartridges and washed with water and then 20% aqueous methanol (*v*/*v*). Bound MMPB was eluted into clean glass tubes with 2 × 1.5 mL of 80% aqueous methanol (*v*/*v*). The eluents were evaporated to dryness under a gentle stream of nitrogen, reconstituted in 200 μL of 50% aqueous methanol (*v*/*v*) and transferred to a microvial for analysis.

### Evaluation of cyanotoxins in fish samples

#### Field sample preparation

Field samples (*n* = 60; 34 fish muscle, 17 livers and 9 egg samples) as well as 50 mL water samples (*n* = 5) were collected from five aquaculture farms across SE Asia. Each fish was gutted and filleted separately with any liver or egg samples found also excised for analysis. All fish samples were lyophilised using a Christ freeze-dryer (Osterode, Germany) to increase the surface area for extraction of the cyanotoxins, allowing for a greater biomass to extraction solvent ratio, enhancing the extraction efficiency. This step enabled the extract to be spun at a higher speed to obtain a cleaner supernatant and to reduce the loading volume and time for the SPE clean-up. The supernatant for SPE must be diluted fivefold, whereby a larger starting mass requires a larger extraction volume and, subsequently, a larger loading volume after dilution, increasing the time to load both cartridges. After lyophilisation, samples were homogenised using a Retsch Ball Mill (Haan, Germany) before storage at −20 °C prior to extraction and analysis. Water samples were lyophilised using a Christ freeze-dryer (Osterode, Germany) and stored at −20 °C prior to extraction and analysis.

#### Analysis of free cyanotoxin content in fish

Samples (100 mg), including liver and eggs, were weighed into a 2-mL tube and spiked with 15 μL of the N^15^-labelled CYN internal standard (N^14^ → N^15^) at 1 μg/mL. Toxin extraction and subsequent enrichment were carried out as outlined above in the “Extraction of cyanotoxins in water and validation of the extraction method in fish” section. In order to quantify any toxins identified, a six-point extracted matrix-matched calibration curve in the range 0.005–0.1 μg/g dw using pooled blank tilapia muscle tissue was prepared as above, with the calibrant levels achieved by spiking with a multi-toxin standard at 0.1 μg/mL along with 15 μL of the N^15^-labelled CYN internal standard (N^14^ → N^15^) at 1 μg/mL.

#### Analysis of total microcystin content in muscle samples

Fish muscle samples (50 mg) were weighed into 50-mL round bottomed flasks with the conversion and subsequent enrichment of MMPB carried out as outlined above in the “Lemieux oxidation and extraction of MMPB” section. In order to quantify any MMPB detected, a seven-point extracted matrix-matched calibration curve in the range 0.025–1 μg/g dry weight using pooled blank tilapia was used, with the levels achieved by spiking with the MMPB standard at 0.5 μg/mL after pH adjustment.

## Results

### UPLC-MS/MS development for total microcystin (MMPB)

Detection and analysis of MMPB, and of MC-LR to confirm complete oxidation, were initially examined using negative polarity (ES^−^) as both analytes can form the [M − H]^−^ ion, although MC-LR was seen as [M − 2H]^2−^. This was achieved using mobile phases comprised of water containing 0.1% acetic acid (*v*/*v*) and acetonitrile and was compared to the analysis of MMPB and MC-LR using positive polarity (ES^+^) with the same mobile phases, as well as changing the additive in the aqueous mobile phase to 0.1% formic acid (*v*/*v*). The sensitivity of the MMPB product ions and, in particular, the qualifying ion (*q*) in negative polarity was too weak, especially in the matrix. The best sensitivity for the analytes was observed by the use of 0.1% acetic acid (*v*/*v*) in the aqueous mobile phase, with ES^+^ chosen as the mode of analysis for MMPB as an [M + H]^+^ ion, whereas MC-LR proved better as an [M − 2H]^2−^ ion.

### Method validation

The method was fully validated to the EC Directive 2002/657 for eight of the nine cyanotoxins, with ATX-A only partially validated due to the presence of the amino acid phenylalanine (Phe). Since these compounds are isobaric and elute similarly, and with Phe naturally occurring in fish at high levels, the amount present in the tissue used for validation generally resulted in the two peaks almost merging into one peak whereby separation and integration was difficult. Consequently, with them sharing transitions, it was difficult to ascertain an accurate CC_α_ (LOD) and CC_β_ (LOQ) for ATX-A. However, in the MS method, a second qualifier ion for ATX-X only was used as a diagnostic ion to prevent misidentification with Phe: *q*1 = 166.1 > 42.95.

As no certified reference materials (CRMs) were used in validating the method and as the added analyte may not act as it does in a real sample, the EC Directive 2002/657 states that at the spiking level of 50 μg/kg (0.05 μg/g) used, the minimum trueness tolerance has to be in the range −20 to +10%. However, it should also be stated that the toxin in real samples may not present for analysis in the same manner as that of the added standard. Similarities in *masking* may arise to those previously described for mycotoxins [31]. The added standard is freely available in the matrix, but toxin in the sample may be covalently bound or masked within the tissue as a non-covalent, associative interaction between toxin and matrix and, unless the extraction process releases the toxin in its free form, it may go undetected. Taking this into account, the resulting validation showed that the method was fit for purpose, with the data outlined in Table 2, including results for linearity (*r* ^2^), absolute recovery, calculated conc., trueness, inter- and intra-day precision analysis, CC_α_ (LOD) and CC_β_ (LOQ). The CC_α_ and CC_β_ values were based on the less intense qualifying ion (*q*) for all analytes, and with an injection volume of only 1 μL, the mean on-column CC_α_ (LOD) and CC_β_ (LOQ) values are 0.9 and 1.5 μg/kg dw (ppb), respectively.

**Table 2:** Results from the validation of the nine cyanotoxins in fish muscle to the EC Directive 2002/657

Analyte	Linearity (*r* ^2^)	Absolute recovery^a^ (%)	Trueness (%)	Inter-day repeatability (rsd %)	Intra-day repeatability (rsd %)	CC_α_ (μg/kg dw^b^)	CC_β_ (μg/kg dw^b^)
ATX-A	0.999	57.7	+1.8	4.2	3.8	–	–
CYN	0.999	99.2^c^	−0.2	2.0	1.7	1.8	2.7
NOD	0.999	83.1	−2	7.7	4.9	0.4	0.8
MC-RR	0.999	87.6	+6.8	4.7	4.4	1.1	1.5
MC-LA	0.997	76.5	−2.8	7.0	6.2	0.6	0.9
MC-LF	0.999	75.2	+7	5.2	4.7	1.4	2.1
MC-LR	0.998	80.3	+3.6	5.7	5.1	0.5	0.8
MC-LY	0.998	76.9	−0.4	8.3	6.5	1.0	1.8
MC-YR	0.999	78.5	−5.2	7.5	4.4	0.7	1.4

The table shows trueness, inter- and intra-day precision analysis (rsd %), linearity (*r* ^2^), absolute recovery, CC_α_ (LOD) and CC_β_ (LOQ)

^a^Absolute recovery is expressed as the extraction efficiency when spiked with 5 ng of toxin per 100 mg lyophilised fish muscle, equating to 50 ng/g (50 μg/kg)

^b^Dry weight (after lyophilisation)

^c^Corrected by the use of CYN (N^14^ → N^15^) internal standard

### Field sample analysis

#### Aquaculture pond water

Analysis of the water from the five aquaculture farms was considered as total toxin load as cells were lysed on lyophilisation and so included both intra- and extracellular toxin. Samples were not filtered on collection as a large proportion of CYN in toxic blooms of *C. raciborskii* has been reported as being extracellular [2]. Out of the five aquaculture farms tested, three (60%) were found to contain cyanotoxins, with two testing positive for both CYN and MC-LR and the third containing CYN only. The levels observed were relatively low (Table 3) with the highest level detected being CYN at a level of 0.27 μg/L in farm 1–1. All concentrations found were below the recommended WHO limit of 1 μg/L set for freshwater [5], with the MC-LR level observed in farm 1–1 similar to that seen across four sampling sites from a Mexican lake (Lago de Patzcuaro) characterised by a persistent cyanobacterial bloom [21].

**Table 3:** Results showing the levels and cyanotoxins found in the water from the aquaculture farms sampled

Farm I.D.	Toxin	
	CYN (μg/L)	MC-LR (μg/L)
1–1	0.27	0.14
1–3	nd	nd
1–4	nd	nd
1–5	0.02	nd
2–1	0.19	0.08

*nd* not detected

#### Determination of free toxin(s) in fish samples

**Fig. 2:**
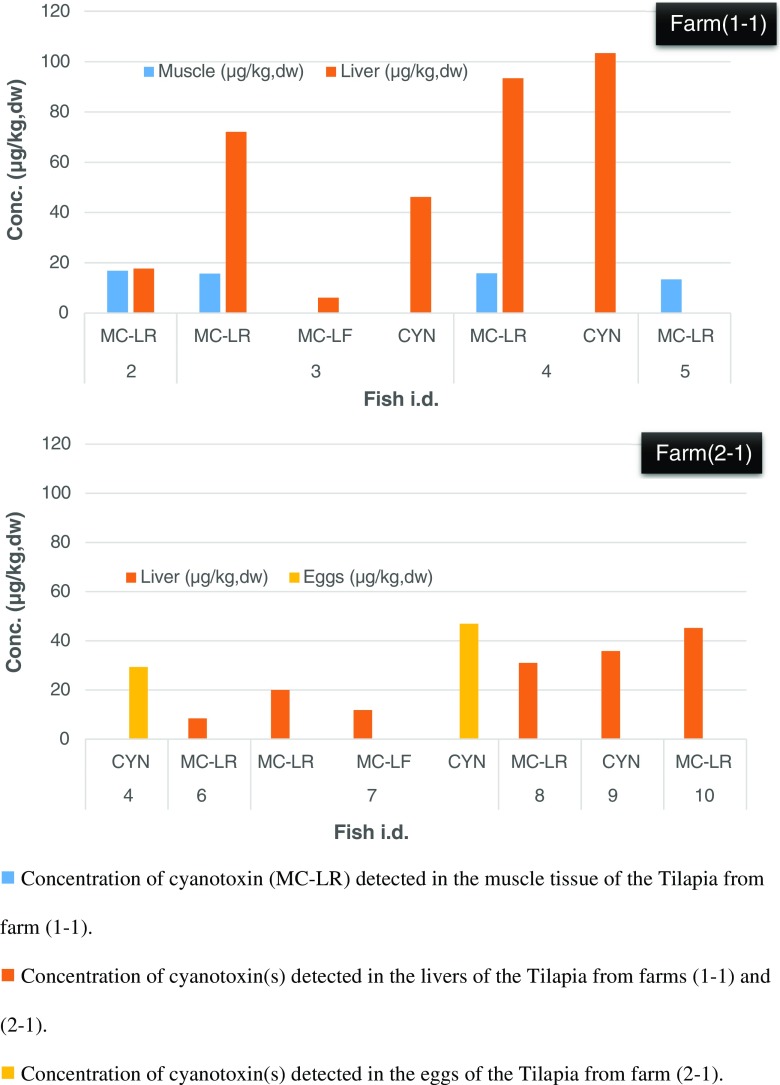
Results of the *free* toxin levels detected in the muscle tissue, liver and eggs of the tilapia harvested from the aquaculture farms

A total of 34 fish (muscle tissue), 17 livers and 9 egg samples were extracted and analysed. A summary of those found to contain cyanotoxins is provided in Fig. 2. As the validation was only performed on fish muscle, the levels characterised in the liver and eggs can only be described as estimates. The results show four fish muscle (12%), eight livers (47%) and two eggs (22%) were found to contain cyanotoxins, MC-LR and MC-LF as well as CYN. Only one cyanotoxin, MC-LR, was identified in the muscle of any of the fish tested, with all the positives found in aquaculture farm 1–1. In this farm, 80% of the tilapia sampled contained MC-LR with the levels detected in the fish muscle ranging from 13.4 to 16.8 μg/kg dw, with a mean level of 15.5 μg/kg dw.

The highest toxin levels identified in the tissues sampled were MC-LR at 16.8 and 45.2 μg/kg dw in the muscle and liver, respectively; MC-LF at 11.8 μg/kg dw in the liver only; and CYN at 103.4 and 46.9 μg/kg dw in the liver and eggs, respectively. None of the fish muscle from farm 2–1 was positive for MC-LR, or any of the cyanotoxins studied, despite the water from this aquaculture farm having low levels of toxins present.

Of the five fish from farm 1–1, three livers were also excised and tested with the integrity of the other two livers compromised. Two of these livers showed high levels of cyanotoxins, with fish 3 containing MC congeners MC-LR and MC-LF, along with CYN, giving a combined estimated toxin load of 124.4 μg/kg dw. A second liver sample from farm 1–1, fish 4 showed the presence of MC-LR and CYN at levels of 93.5 and 103.4 μg/kg dw, respectively, giving a combined estimated toxin load of almost 200 μg/kg dw.

Of the 19 fish harvested from farm 2–1, 11 had their livers and/or eggs also removed and tested as a representative sample, with the other livers compromised by being stored frozen. Over 70% of the livers tested from farm 2–1 contained CYN and MC congeners MC-LR and MC-LF, with the highest levels detected of each estimated at 46.9, 45.2 and 11.8 μg/kg dw, respectively. This analysis also identified CYN in the eggs of two fish from farm 2–1, with levels of 29.3 and 46.9 μg/kg dw for fish 4 and 7, respectively.

#### Determination of total MC levels (free plus bound/conjugated)

**Fig. 3:**
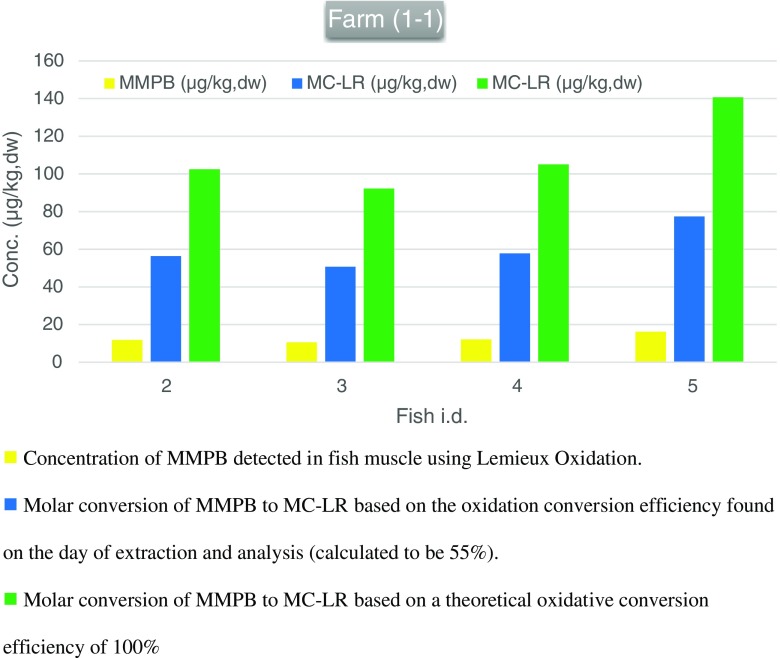
Results of the MMPB detected in fish from aquaculture farm 1–1 with the molar equivalents of MC-LR calculated

The results of the MMPB analysis (Fig. 3) showed the same four fish from aquaculture farm 1–1 contained MMPB, with the levels detected ranging from 10.6 to 16.2 μg/kg dw and a mean estimated value of 13.0 μg/kg dw. These levels account for the total level of MC present, i.e. protein-bound plus free MC. On conversion of the amount of MMPB observed to molar equivalents of MC-LR (100 ng MC-LR forms 21 ng MMPB assuming 100% oxidation conversion), the level of MC-LR (or equivalents) detected equated to 50.7–77.4 μg/kg dw. These values are based on an oxidation conversion efficiency of 55% as calculated on the day of extraction by the use of a control experiment, calculated by spiking control samples with MC-LR (100 ng) before oxidation to samples spiked with the corresponding amount of MMPB (21 ng) after oxidation. Accounting for losses due to incomplete oxidative conversion and calculating back to molar equivalents of MC-LR present, the levels range from 92.2 to 140.7 μg/kg dw, with a mean estimated value of 110.1 μg/kg dw.

#### Determination of *bound* microcystin present

With an average of 15.5 μg/kg dw of free MC-LR identified in the fish muscle and a mean estimated value of 110.1 μg/kg dw of total MC present in the same tissue, the estimated mean level of conjugated MC present in the muscle tissue equates to 94.7 μg/kg dw. Overall, the amount of conjugated or *masked* MC present accounts for 85% of the total.

#### Bioaccumulation of MC in fish tissue

Farm 1–1 shows the presence of MC-LR in the water body at 0.14 μg/L and in the muscle of the fish at an average level of 15.45 μg/kg; therefore, the average bioaccumulation factor (BAF) taken at the time of harvest equates to 110, with a BAF of >1 indicating bioaccumulation [32].

Taking the livers from farm 1–1, fish 2 shows a BAF of 126 for MC-LR, fish 3 has BAFs of 515 and 171 for MC-LR and CYN, respectively, whereas fish 4 shows BAFs of 667 and 383 for MC-LR and CYN, respectively. In comparison to fish livers taken from aquaculture farm 2–1 where the water was found to contain both MC-LR and CYN at levels of 0.08 and 0.19 μg/L, fish 6, 7, 8 and 10 indicate BAFs of 105, 250, 387 and 565, respectively, for MC-LR, with fish 9 indicating a BAF of 188 for CYN.

#### Conversion of values from dw to ww

The level of toxin reported in the tilapia (*O. niloticus*) samples that were determined to be positive for free MC-LR in fish muscle from farm 1–1 ranged from 13.4 to 16.8 μg/kg dw, with a mean value of 15.5 μg/kg dw. As around 70% of fish tissue contains water and using a conversion factor of 0.31 to convert from dw to ww [33], this gives an average level of 4.8 μg/kg ww. The average total level of MC present as estimated by the use of Lemieux oxidation was 110.1 μg/kg dw, which, when converted, equated to a value of 34.1 μg/kg ww, illustrating an estimated level of conjugated or masked MC of 29.3 μg/kg ww in the muscle tissue.

On evaluating the data generated from aquaculture farm 1–1, the level of MC-LR present in the water was 0.14 μg/L, with the mean level of free MC-LR in fish muscle from this farm being 4.8 μg/kg ww, indicating that at the time of harvest, the fish there has a BAF of 34 [32]. It could be estimated that the total level of MC present in the *O. niloticus* at the time of harvest was 34.1 μg/kg ww accounting for masked microcystins, whereby the BAF would increase to 243.

## Discussion

Several UPLC-MS/MS (or LC-MS/MS) methods have been published for the analysis of cyanotoxins in fish tissue, with these mainly determining MCs, NOD, CYN and ATX-A separately [19, 22, 23, 34–38]. However, to date, there have been no validated UPLC-MS/MS methods published for the determination of all the aforementioned cyanotoxins in fish muscle tissue, across three different classes of cyanotoxin, along with their combined extraction and sample enrichment, making this method novel.

An improvement over other published methods is performing the extraction and analysis in 1 day by the use of 75% aqueous methanol (*v*/*v*) (0.5% acetic acid *v*/*v*) as the extraction solvent in place of BuOH/MeOH/H_2_O (1:4:15) [23]. Extraction with 75% aqueous methanol (*v*/*v*) (0.5% acetic acid) gave acceptable recoveries of the suite of cyanotoxins in comparison to BuOH/MeOH/H_2_O (1:4:15). Taking into account the extraction with BuOH/MeOH/H_2_O (1:4:15) is usually performed over a total of 72 h (3 × 24 h extractions) whereas the extraction with 75% aqueous methanol (*v*/*v*) (0.5% acetic acid *v*/*v*) took only 45 min, it was decided to use the latter to develop the method, saving quite a bit of time.

The results from the analysis of the water samples from the five aquaculture farms showed the presence of cyanotoxins, with levels lower than those reported in studies carried out in Zaria, Northern Nigeria [20], and China [13, 19], but similar to levels reported from a freshwater lake in Mexico [21]. No reports outlining the presence of CYN in aquaculture systems have been identified to date; however, this cyanotoxin was found in 60% of those sampled in the present study. The water samples taken from the five aquaculture farms were all obtained at the time of harvesting the fish, and no further information was provided as to when (if) HABs had occurred. It is, however, likely that farm 1–1 had a bloom in its senescent phase, allowing the fish more time to bioaccumulate released toxin.

Four of the five fish sampled from farm 1–1 accumulated MC-LR, the most acutely toxic variant of the MC congeners. Based on the WHO tolerable daily intake (TDI) value for MC-LR of 0.04 μg/kg body weight per day [5], the estimated daily intake (EDI) of MCs for an adult weighing 60 kg and consuming 300 g of edible organs of aquatic animals equates to 2.4 μg MC-LR per day for safe consumption. Taking the mean level of MC-LR found in the edible portion of the *O. niloticus* at 15.45 μg/kg dw, the exposure from this equates to 4.65 μg MC-LR, almost twice the level deemed safe by the WHO. Taking the estimated mean level of 110.1 μg/kg dw of total MC detected, based on the amount of MMPB detected and corrected for losses due to oxidative conversion, this gives a mean EDI of 33 μg MCs present in the *O. niloticus*. Based on the TDI for MC-LR calculated above, this value is close to 14 times greater and, as such, constitutes a severe health risk if consumed.

On evaluating the levels reported in dw for free MC-LR and converting to ww using a conversion factor of 0.31 [33], the EDI value for the edible portion of the *O. niloticus* equates to 1.4 μg, lower than the safe consumption level suggested by the WHO of 2.4 μg MC-LR. However, considering the total amount of MC present as estimated by the amount of MMPB detected and the conversion to ww using the same coefficient factor of 0.31, the EDI for consumption of muscle tissue in the *O. niloticus* from this study equates to 10.2 μg, 4.25 times higher than that deemed safe by the WHO.

In relation to the estimated level of 29.4 μg/kg ww (94.7 μg/kg dw) of conjugated or masked MC calculated in the muscle tissue (“Determination of *bound* microcystin present” section), it must be noted that the extra MC detected by Lemieux oxidation may not be exclusively from protein-bound MCs through their interaction with PP1 and PP2A as stated previously [26]. Instead, the extra MC(s) detected may be from congeners the method does not detect, with over 100 characterised to date [3], as well as from protein-bound MC. Although the other congeners which may be present may not be as toxic as MC-LR, they are still likely to pose a health risk to consumers and could lead to synergistic effects between different congeners, potentially increasing the toxicity [11]. It must also be considered that these values are based on adult consumption so the exposure is likely to be much more dangerous for children. Furthermore, BAFs calculated show bioaccumulation in the muscle and liver with both tissues having BAFs of >1. The livers show BAFs of up to six times higher than that of the muscle which is expected with it being the target organ; however, as the method was not validated for liver tissue, this is only an estimate.

A study conducted by Jia et al. [23] reported that two omnivorous fish species harvested from Lake Taihu in China contained three MC congeners: MC-LR, MC-YR and MC-RR with combined levels of 26.7 and 31.7 ng/g dw, higher than the mean level found in the muscle of the present study. However, they found MC-RR to be the most prevalent (60–100%) in the samples tested. The level of MC-LR detected in the present study was approximately 5-times higher than that of Jia et al. [23], and with the LD_50_ in mice for MC-RR being approximately 10-fold higher than for MC-LR [5, 39], the toxicity of the *O. niloticus* from this aquaculture farm would be substantially higher. The literature also shows that differing levels and types of MCs have been found in different species of farmed fish [23, 35], with the omnivorous *Carassius auratus* in the study by Zhang et al. [35] showing MCs in the muscle tissue with a maximum value of 13 μg/kg dw and an average of 2.4 μg/kg dw, just over six times lower than that found in the *O. niloticus* of the present study. Again, the majority of MC detected in the muscle of the *C. auratus* was MC-RR, whereas that detected in the *O. niloticus* from this study was MC-LR.

Research conducted by Poste et al. [33] looked at several fish species, including *O. niloticus*, across several tropical (Ugandan) and temperate (North American) freshwater lakes. They detected MCs in the muscle tissue of *O. niloticus* harvested from a number of these lakes, showing an average level of 11 μg/kg ww, just over twice that found in the *O. niloticus* from our study when converted to ww. In their study, detection was performed by ELISA using a MC-Adda antibody, meaning that the individual congeners present could not be elucidated, and as such, the toxicity of these fish may not be as high due to some congeners having a higher LD_50_ value than MC-LR [33]. A further study carried out by Gurbuz et al. [40] also showed accumulation of MCs in the muscle and liver tissue in two omnivorous fish species, *Cyprinus carpio* and *Carassius gibelio*, with one of the fish species, *C. carpio*, showing significantly higher concentrations of MC in the liver over the muscle, similar to that seen in our study, whereas no significant difference was found between tissues in *C. gibelio* [40].

All of the above reports, including those conducted by Zhang et al. [35], Magalhaes et al. [22] and Chen et al. [34] all looked at free MC levels and did not evaluate the level of total MC present. It has been reported that the potential amount of covalently bound MCs can account for between 38 and 99% of the total MC present in an organism’s tissue. With this study showing up to 85% of the MC in the muscle tissue of the farmed fish was possibly conjugated or masked, the total levels present and subsequent exposure reported could be significantly higher. One caveat of this is that covalently bound MC may not be as readily bioavailable and therefore not be as toxic. One study by Smith et al. [41] looked at MC-phosphatase *peptides* produced by proteolytic digestion, mimicking the human gut. They concluded that these products were less toxic in vitro [41]. Importantly though, they were still deemed toxic and would only add to the toxicity conferred by any free MC present.

The study conducted by Chen et al. [13] on fishermen living off Lake Taihu showed MC in the muscle of 16 aquatic species and in the serum of the fishermen, with the proportions of the MC congeners in the blood matching that in the muscle of the fish. This also led to liver damage in these men with their liver function enzymes elevated. This demonstrates that eating fish contaminated with MC will result in the uptake of the toxin, with the health risks relatively unknown apart from the growing links to liver and colorectal cancers [7, 15].

Liver tissue contained the highest amount of toxin in the tissues tested, with estimated levels of 45.2 and 103.4 μg/kg dw for MC-LR and CYN, respectively. This is not surprising considering both MC-LR and CYN are considered hepatotoxic, with the liver being the primary target organ. As *O. niloticus* like many other fish may also be consumed whole [32], the levels that an individual could be exposed to will be substantially higher than for consumption of the muscle only, especially taking into account the levels seen in the livers of the *O. niloticus* from this study, and could constitute a severe health risk to human health.

This is also the first report of the presence of CYN in the eggs of fish, identified in two fish from farm 2–1, with estimated levels of 29.3 and 46.9 μg/kg dw for fish 4 and 7, respectively. This may have consequences for the development and viability of embryos and/or the fry if passed on during reproduction [42], not to mention the economic consequences to the farmer.

Furthermore, a number of livers and eggs from farms 1–1 and 2–1 showed the presence of another peak in the same retention window as MC-YR; however, the exact retention time and ion ratio (*q*/*Q*) were not the same as those of the MC-YR standard. This points to the possibility of the presence of a further MC analogue, MC-RY, recently identified in a cyanobacterial bloom from Lake Victoria, Tanzania. This MC congener, MC-RY, has been reported in freshwater systems in African countries such as Uganda, Kenya and Tanzania [43], with this study indicating its potential presence in fish in SE Asian aquaculture.

## Conclusion

A method was developed and validated for the measurement of nine cyanotoxins in fish muscle tissue, also used to measure free cyanotoxin levels in fish liver and eggs, from a number of typical pond-based aquaculture farms in SE Asia. In one of the farms studied, the levels of free MC-LR present were lower than the level deemed safe by the WHO. When the total MC level is taken into account, the toxin present was on average around four times higher than the recommended WHO TDI, indicating that a substantial proportion of MCs are masked and that the levels reported in the literature are more than likely vastly underestimated. Even though the levels of free MC-LR found indicate that the fish are safe to eat, the levels of total MC estimated from this study show the opposite. These data do, however, indicate that consumption of such contaminated fish constitutes a severe health risk, especially if eaten whole due to the levels detected in the fish livers. There is also the possibility that consumption of fish contaminated with MC-LR could lead to liver cancer due to chronic long-term exposure. With climate change and eutrophication on the increase globally, the subsequent rise in HABs across freshwater systems which are increasingly used for aquaculture, the potential for large-scale contamination of fish by freshwater cyanotoxins raised in aquaculture systems appears likely. Those who are of particular concern are fishing communities, whereby the consumption of water and fish from fresh water bodies subjected to dense HABs constitutes a health risk due to chronic exposure year-round from MCs and other cyanotoxins. This study indicates that further monitoring and control of the effects of HABS and cyanotoxins on the safety of farmed fish is required and this may lead to the need for regulations to be put in place in aquaculture systems. There also appears the need for total microcystin levels to be quantified due to the majority of the microcystins present in the fish being masked, similar to mycotoxins where food safety legislation is being rethought due to this issue.
